# “A Mother Makes a Better Nurse”: A Phenomenological Study of Neonatal Nurses' Readiness Recalibration Following Postpartum Return to Work

**DOI:** 10.1155/jonm/9095340

**Published:** 2025-11-02

**Authors:** Yuan Li, Zongrong Zhou, Mei Rosemary Fu, Jun Tang, Hanmei Peng, Liyu Zhu, Chuanya Huang, Qiong Chen, Yanling Hu

**Affiliations:** ^1^Department of Nursing, West China Second University Hospital, Sichuan University/West China School of Nursing, Sichuan University, Chengdu, China; ^2^Key Laboratory of Birth Defects and Related Diseases of Women and Children (Sichuan University), Ministry of Education, Chengdu, China; ^3^Department of Neonatology, West China Second University Hospital, Sichuan University, Chengdu, China; ^4^Department of Reproductive Medicine Nursing, West China Second University Hospital, Sichuan University, Chengdu, China; ^5^School of Nursing and Health Studies, University of Missouri-Kansas City, Kansas City, Missouri, USA; ^6^West China School of Nursing, Sichuan University, Chengdu, China; ^7^Department of Obstetrics and Gynecology Nursing, West China Second University Hospital, Sichuan University, Chengdu, China

**Keywords:** lived experience, neonatal nurse, postpartum period, qualitative research, return to work

## Abstract

**Aim:**

To explore the lived experience of neonatal nurses returning to work postpartum, focusing on their adaptation processes and the recalibration of professional readiness under the influence of motherhood.

**Background:**

The transition back to work postpartum for nurses, particularly in high-acuity areas like neonatal care, presents multifaceted challenges. While existing literature addresses general nurses' return-to-work experiences, a significant gap exists in understanding the specific lived experience of neonatal nurses. Moreover, the potential for maternal identity to reshape professional practice remains underexplored.

**Methods:**

A descriptive phenomenological study was conducted. Using a purposive sampling strategy, seventeen neonatal nurses who returned to work within 1–12 months postpartum in China were recruited. Data were collected through individual in-depth, semistructured interviews and analyzed using Colaizzi's seven-step method.

**Results:**

Four interconnected themes emerged revealing a dynamic process of “readiness recalibration” that demonstrates how “a mother makes a better nurse”: (1) establishment of maternal identity fundamentally reoriented life priorities and emotional frameworks; (2) multifaceted adaptation challenges encompassed prereturn anxiety, physiological and psychological impacts, skill readjustment, and breastfeeding–work integration difficulties; (3) maternal identity–enhanced empathetic capacity, transformed care behaviors, and strengthened family-centered care philosophy while creating dual role conflicts; and (4) ecosystem factors either facilitated or constrained successful transition with significant institutional support gaps identified.

**Conclusion:**

The postpartum return for neonatal nurses represents a profound journey of professional enhancement, not merely recovery. Maternal experience serves as an informal education that enriches neonatal care, but this transition requires robust institutional support to mitigate unique challenges and optimize nurse well-being.

**Implications for Nursing Management:**

Healthcare organizations should implement comprehensive support strategies, including structured re-entry programs, robust breastfeeding accommodation, and flexible scheduling, to harness the enhanced capacity motherhood brings while addressing the unique vulnerabilities and adaptation challenges of returning neonatal nurses.

## 1. Background

The postpartum return to work represents a pivotal and often strenuous life event for working mothers, characterized by multifaceted physiological recovery, psychological adjustment, and identity renegotiation that profoundly impact their professional readiness [1–3]. This transitional period is marked by significant vulnerability, as many women are still navigating the lingering effects of childbirth [4, 5]. Specifically, the postpartum period brings physical recovery challenges, persistent fatigue, and hormonal fluctuations that can trigger negative emotions like anxiety and distress, with many of these symptoms often lasting a year or more beyond delivery [6–8].

For nurses, postpartum return-to-work challenges are significantly amplified due to inherent occupational stressors, such as inflexible schedules, shift work, and high emotional labor, which intersect directly with their personal recovery and new maternal responsibilities [8, 9]. The resulting stress is substantial; for example, a cross-sectional study of returning nurses in China found that 60.27% experienced moderate-to-severe work-related stress [10]. This intersection of personal vulnerability and professional demands creates a particularly complex dynamic especially high-acuity settings [10, 11]. Consequently, successfully navigating this transition is not only a personal matter but also a critical issue for the healthcare system [12, 13]. Indeed, poor adaptation among returning nurses has been linked to increased burnout, higher turnover intentions, and potential compromises in the quality and safety of patient care [8, 9, 12].

The neonatal care environment presents a unique setting for this transition. Neonatal nurses operate in a high-stakes setting, providing sophisticated care to vulnerable and critically ill newborns, which requires advanced technical skills and profound emotional engagement [14]. When these nurses return to work as new mothers themselves, they encounter a powerful emotional resonance while caring for infants of similar age and vulnerability as their own children [15]. This direct intersection of their nascent maternal identity and professional duties creates a uniquely specialized and emotionally charged experience that distinguishes them from other specialty nurses [12, 16] and necessitates dedicated investigation.

While a growing body of literature addresses the return-to-work experiences of nurses [8], research has largely focused on nurses in general [9–13, 16]. These studies identify common challenges, such as work–family conflict, inadequate supervisor support, breastfeeding obstacles, and childcare difficulties [8, 9, 17, 18]. However, two significant gaps remain. First, none of the studies have focused on neonatal nurses, whose unique experiences are often diluted when aggregated with broader nursing groups. Second, the literature is predominantly deficit-oriented, framing the postpartum return almost exclusively as a period of conflict and struggle [2, 6, 16]. This perspective overlooks the potential for positive transformation—specifically, how maternal identity can influence and reshape professional practice, enhancing empathetic capacity, and change caregiving behaviors [19]. The process of how this dual identity of mother–nurse is negotiated and leads to “readiness recalibration” remains poorly understood.

The global nursing workforce has grown to 29.8 million by 2023, with women comprising 85% of this essential healthcare workforce and approximately one-third under the age of 35 [20], making the postpartum return to work a pivotal workforce issue for healthcare systems worldwide. In China, this reality is even more pronounced: women constituted 98.7% of the 5.63 million registered nurses, and 60.3% of this group were in their prime childbearing years by the end of 2023 [21, 22]. China's national three-child policy, introduced in 2021, further amplifies this challenge, suggesting that a substantial and growing proportion of these nurses will face the postpartum return to work [23]. Furthermore, the unique sociocultural landscape in China—characterized by traditional family values and societal expectations of motherhood—adds additional complexity to these nurses' experiences [24, 25]. These demographic and contextual realities underscore postpartum return to work as a central challenge for workforce stability and care quality both globally and within the Chinese healthcare context. Therefore, understanding postpartum return-to-work experiences is crucial for healthcare organizations worldwide to develop effective support strategies that can enhance nurse well-being, improve retention rates, and ensure the quality of care provided to the most vulnerable patients in neonatal units.

This study utilized a descriptive phenomenological approach to gain a deep understanding of the lived experience of neonatal nurses returning to work postpartum in China. The research objectives were to (1) explore the adaptation processes of neonatal nurses returning to work postpartum, examining their physical, emotional, and professional readiness; (2) describe how maternal identity influences and reshapes nursing practice and caregiving behaviors; and (3) identify both facilitating and constraining factors in the postpartum return-to-work experience to inform evidence-based organizational interventions.

## 2. Methods

### 2.1. Study Design

This study employed a descriptive phenomenological design [26–28] to explore the lived experience of neonatal nurses returning to work postpartum. Descriptive phenomenology was considered optimal because it enables researchers to capture the essence and meaning of shared human experiences while bracketing presuppositions to allow the phenomenon to emerge naturally [26]. This approach was particularly suited for gaining a deep understanding of the unique experience of postpartum return to work through direct description of participants' perceptions, feelings, and interpretations of their return-to-work journey. The study design and reporting followed the Standards for Reporting Qualitative Research (SRQR) guidelines [29] (Supporting Table 1).

### 2.2. Sample and Setting

Participants were recruited from three neonatal units at West China Second University Hospital in Chengdu, Sichuan Province, including Neonatal Intensive Care Units and general neonatal wards. Purposive sampling combined with a maximum variation strategy was used to ensure sample diversity across educational background, neonatal nursing experience, delivery mode, and maternity leave duration. Sample size was determined by the principle of data saturation [28, 30, 31], which was considered achieved when three consecutive newly recruited participants provided no new themes or significant information.

Inclusion criteria included registered nurses who (i) worked in neonatal care; (ii) returned to work within the past 1 to 12 months following childbirth; (iii) had minimum 2 years' experience in current unit before maternity leave; (iv) were engaged in direct clinical care for at least 3 days per week; and (v) were able to articulate experiences in Mandarin Chinese and willing to participate. Exclusion criteria included registered nurses who (i) experienced major health complications (e.g., severe postpartum depression and significant physical issues) or (ii) encountered significant family upheavals (e.g., bereavement, marital separation, or serious illness of a partner) during their maternity leave. These exclusion criteria were established to focus on the typical postpartum return-to-work experience, as conditions like major health complications or family upheavals represent significant confounding factors that warrant separate investigation.

### 2.3. Recruitment and Data Collection

Recruitment was facilitated through nurse managers who identified potentially eligible nurses within their units and obtained consent to be contacted. Researchers subsequently contacted potential participants, provided comprehensive study information, obtained written informed consent, and scheduled interviews at times and locations convenient for participants. One day prior to each interview, participants received an electronic questionnaire via the REDCap system to collect demographic data and an interview reminder.

Data were collected through individual face-to-face in-depth interviews conducted by a trained qualitative researcher (Z.Z.R.) between March 2025 and May 2025. The interviewer had no prior contact with participants. The entire research team maintained bracketing through reflective diaries to set aside personal, professional, and cultural assumptions about postpartum experiences, including preconceptions related to Chinese ideals of motherhood and family roles [28, 31]. Interviews were conducted in quiet, private environments to ensure authentic expression. Interviews lasted between 50 and 75 min. With participants' consent, all interviews were audiorecorded and transcribed verbatim within 24 h, yielding a total of 241,700 Chinese characters for analysis. Field notes capturing nonverbal cues and researcher reflections were taken to enrich the data.

#### 2.3.1. Interview Guide

A semistructured interview guide was developed based on research objectives and literature review and then refined through team consensus. The guide featured open-ended questions designed to encourage rich descriptions of participants' feelings, perceptions, and attributed meanings related to their return-to-work experiences, with probing questions used to explore topics in greater depth (Supporting Table 2). The guide was pretested with two eligible individuals who were not included in the main study to ensure study integrity.

#### 2.3.2. Research Team

The research team consisted of four primary researchers and five research assistants, including five doctoral-level experienced nursing and neonatal experts with qualitative research expertise. Primary researchers provided methodological leadership in phenomenological inquiry, study design, and data collection and analysis. While interviews were conducted by one trained researcher, the team collectively contributed to interview guide development, bracketing processes, and interpretive validation. Research assistants facilitated data management and transcription.

### 2.4. Data Analysis

Data analysis was performed concurrently with data collection, following Colaizzi's seven-step phenomenological analysis method [32]. Two primary researchers (L.Y. and Z.Z.R.) independently conducted the initial analysis. The process began with familiarization by repeatedly reading the transcripts to achieve full immersion and gain a holistic sense of each participant's account (Step 1). Significant statements directly pertaining to the return-to-work phenomenon were systematically extracted (Step 2), followed by independent formulation of meanings that captured participants' essential messages while preserving authenticity (Step 3). Discrepancies were resolved through collaborative discussion. Member checking was conducted after this step, where formulated meanings were returned to participants for validation, leveraging their fresh recall to yield valuable clarifications [33]. Validated meanings were then clustered into themes (Step 4), synthesized into exhaustive descriptions supported by verbatim quotes (Step 5), and distilled into fundamental structures of the phenomenon (Step 6). Finally, the overall analytical framework was validated through team consensus and consultation with external experts in neonatal nursing and qualitative research (Step 7). NVivo 15.1.3 (QSR International Pty Ltd, Massachusetts, USA) was used for data management and analysis.

### 2.5. Rigor and Trustworthiness

The rigor and trustworthiness of this study were established according to Lincoln and Guba's criteria [34]. Credibility was enhanced through multiple strategies: member checking, data triangulation (integrating interview data with field notes and reflective diaries), independent analysis by two researchers, prolonged engagement with participants, and verification of data accuracy by listening to each interview recording while checking against transcripts. Dependability was established through a comprehensive audit trail, consistent data collection by a single trained investigator using a piloted guide, and an external audit by a qualitative research expert. Confirmability was addressed through sustained reflexivity and grounding all themes in verbatim participant quotations. Transferability was facilitated by providing thick descriptions of the participants, setting, and findings to allow readers to assess applicability to other contexts.

## 3. Results

### 3.1. Demographic Characteristics of Participants

Eighteen eligible neonatal nurses were invited to participate; one withdrew shortly before the interview due to personal health reasons, resulting in a final sample of seventeen participants. The participants had a mean age of 30.18 years (SD = 2.43) and averaged 6.35 years of neonatal nursing experience (SD = 2.23). Most participants (82.35%) were first-time mothers. The youngest children averaged 9.12 months of age (SD = 2.98). The majority (64.71%) delivered via cesarean section. Upon returning to work, a significant portion (76.47%) continued breastfeeding, and while most participants (70.59%) reported having access to some form of private space for lactation, only a minority (23.53%) reported having access to a dedicated lactation room. A detailed summary of participant demographics is presented in Table 1 and Supporting Table 3.

### 3.2. Core Themes of the Lived Experience

**Figure 1:**
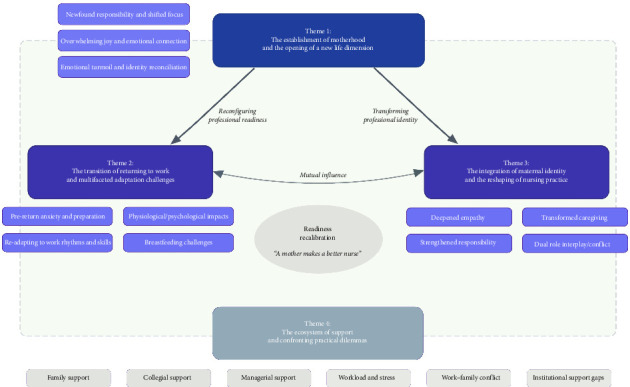
Thematic map of lived experience of neonatal nurses returning to work postpartum.

Through systematic analysis guided by Colaizzi's seven-step method, four core themes emerged to illuminate the essential structures of neonatal nurses' postpartum return-to-work experience as illustrated in Figure 1. These themes revealed a dynamic process of readiness recalibration that demonstrates how “a mother makes a better nurse,” as nurses navigated between maternal and professional identities: the establishment of maternal identity (Theme 1) fundamentally reconfigured existing professional readiness, necessitating a multifaceted transition period (Theme 2), during which maternal identity integrated with and enhanced nursing practice (Theme 3), all within an ecosystem that either facilitated or constrained this recalibration process (Theme 4). This recalibration process ultimately illustrated that maternal experience served as an informal education for professional development, enriching nursing competence.

#### 3.2.1. Theme 1. The Establishment of Motherhood and the Opening of a New Life Dimension

This theme captured the foundational reconfiguration of nurses' existing identity as they embraced motherhood, initiating the readiness recalibration process. Three subthemes revealed how the establishment of maternal identity altered their life orientation: newfound responsibility and shifted focus, overwhelming joy and emotional connection, and emotional turmoil and identity reconciliation.

##### 3.2.1.1. Newfound Responsibility and Shifted Focus

The emergence of maternal identity reconfigured participants' life priorities and sense of responsibility. P1 stated “*I feel my responsibility is heavier… when I get off work, the first thing I think about is going back to take care of him [her baby]*.” This sentiment was epitomized by P11, who described her child as a new anchor in life: “*I feel like I had no emotional anchor before, but now I have one. She has truly become my anchor. All my attention is now focused on her*.” P16 further illustrated this shift: “*After having a baby, my own time is basically occupied by the baby… all my energy is given to him*.” This reorientation of life's focus formed the new personal baseline from which professional readiness would be recalibrated.

##### 3.2.1.2. Overwhelming Joy and Emotional Connection

The emotional transformation accompanying maternal identity establishment created new emotional resources. P17 expressed “*Seeing him [my baby] or holding him, you feel like you own the world…watching him grow a little bit every day, I also feel a sense of accomplishment*.” For some, this connection enhanced family relationships, as P9, who had longed for a child, shared, “*My child was very much wanted…after he was born…I feel my husband also quite likes the child, and the family is more harmonious, I feel very happy*.” P5 captured the essence of maternal fulfillment as being “*tired but happy*,” adding that “*happiness is definitely having a tiny life in your hands, in your arms, and feeling such contentment*.” This deep emotional enrichment subsequently influenced their professional nursing practice.

##### 3.2.1.3. Emotional Turmoil and Identity Reconciliation

The establishment of maternal identity was not seamless, creating identity conflicts and internal tensions. P2 candidly shared “*I even felt a bit regretful, feeling that having a baby cost me too much, my whole life was messy and frustrating*.” P12 articulated the tension between maternal demands and personal autonomy: “*I still want to be myself a bit…I don't want my whole life to be completely filled with children*.” However, these identity conflicts were typically resolved through maternal connections, as P6 (mother of twins) described: “*For about 2 days, I didn't want to see them…but he looked at me and smiled, and then I got better*” (P6S4). These accounts highlighted the intense emotional adjustment and identity reconciliation required in early motherhood.

#### 3.2.2. Theme 2. The Transition of Returning to Work and Multifaceted Adaptation Challenges

This theme detailed the challenging transition period as nurses moved from established maternal identity back to professional environments. The challenges encountered across multiple domains demonstrated the complex nature of readiness recalibration, encompassing four key areas: prereturn anxiety and preparation, physiological and psychological impacts, readapting to work rhythms and skills, and breastfeeding and work integration challenges.

##### 3.2.2.1. Prereturn Anxiety and Preparation

Anticipation of returning to work was often accompanied by anxiety, largely stemming from concerns about inadequate professional readiness. P4 (maternity leave for 12.7 months) described her prereturn state: “*I was definitely anxious because I hadn't been working for so long…I might not do well in my job… and my memory felt super bad after giving birth*.” This lack of confidence in professional readiness was a common concern. To cope, nurses engaged in various preparations. P15 mentioned “*Regarding important operational procedures…I reviewed them at home before returning to work to get ready*.” Family arrangements were also crucial; P9 detailed “*I had my mom come a month in advance to adapt to feeding him (the baby)…after she got the hang of it, I felt very relieved*.” Practical and emotional preparations, including ensuring childcare arrangements, were reported as essential for building readiness.

##### 3.2.2.2. Physiological and Psychological Impacts

The initial weeks back at work were often physically and mentally taxing. P1 reported “*I had persistent back pain after childbirth…my shoulders and neck would also hurt*.” P15 experienced physical discomfort due to the new work environment: “*The lights of the new ward are very strong, I felt dizzy and my head was spinning all day.*” Cognitive challenges, often termed “*baby brain*,” were also prevalent. P12 shared “*My memory is very poor, on the first day I could clearly feel I couldn't remember things,*” while P13 experienced similar difficulties: *“I would forget things immediately after being told*.” These internal strains were compounded by a heightened psychological vulnerability, manifesting as overwhelming emotional reactions when encountering distressing patient conditions. This was poignantly described by P15, who cared for a baby with Treacher Collins Syndrome (characterized by bird-like facial features): “*I had never been scared by a baby's appearance before, but seeing him, I was truly terrified…It wasn't just pity; it brought me a sense of dread.*” This reaction appeared to suggest heightened maternal vulnerability and projective anxiety about their own child's well-being.

##### 3.2.2.3. Readapting to Work Rhythms and Skills

Professional readiness required active reconstruction of technical competencies and workflow adaptation. P15 described the skill recovery process: “*I was worried about my injection and blood drawing skills…but after trying, I found that muscle memory was still there*.” P9 reflected on growth through the transition: “*After that first night shift…I felt I had grown…I had relearned things I seemed to have forgotten*.” However, environmental and technological changes complicated readiness adjustment. P17 encountered significant barriers: “*The ultrasound machine model had changed…I wasn't familiar with the new equipment…each ultrasound-guided PICC insertion took 10 to 20 min, which was too time-consuming*”. These experiences demonstrated how readiness adjustment extended beyond personal adaptation to include environmental refamiliarization.

##### 3.2.2.4. Breastfeeding and Work Integration Challenges

For participants who continued breastfeeding (52.94% exclusively, 23.53% mixed feeding), integration with work presented significant challenges to daily functioning and well-being. P8 found it “*very troublesome*” due to the logistics of pumping. P17 reported temporal conflicts: “*If I'm busy, I won't have time to pump milk*.” Despite institutional policies allowing lactation breaks, implementation was often difficult, as flexible timing based on unit workload created practical barriers. P10 described a painful resolution: “*Although permitted, my work couldn't accommodate pumping time because it was too busy…You can't have it both ways, you have to choose one, so I had to let my child down a bit*.” P17 further described the tension: “*As a new mother, you worry about not having enough milk, but clinical work is indeed very busy, and sometimes it affects your milk supply*.” This perceived lack of practical support was a direct impediment to navigating their dual identity demands.

#### 3.2.3. Theme 3. The Integration of Maternal Identity and the Reshaping of Nursing Practice

Most critically, this theme revealed how maternal identity reshaped professional practice, providing evidence for the central finding that “*a mother makes a better nurse*.” This was evident through the subthemes of deepened empathy and compassionate engagement, transformed care behaviors and approaches, strengthened responsibility and evolved care philosophy, and interplay and conflict of dual roles.

##### 3.2.3.1. Deepened Empathy and Compassionate Engagement

Maternal identity elicited profound empathy, reshaping emotional responses in patient care. P14 described this transformation: “*After becoming a mom myself, I feel more overflowing maternal love toward these babies…I want to protect them more*.” This enhanced empathy manifested as embodied, visceral responses that drove nurses toward more active compassionate engagement. P5 described “*When drawing blood from a baby and he's wailing, my heart aches as if I'm being stabbed. I literally break out in a sweat…It feels like their pain is my own. I find myself staying longer to comfort them*.” The deepened empathy extended to a deeper understanding of neonatal patients' families through shared maternal experience. P6 reflected “*Seeing a mother who had just given birth crying, I could really empathize with [her]…because when my own child was hospitalized, I also cried*.” This shared understanding prompted more intentional emotional support, as P6 noted “*Now I actively spend extra time reassuring anxious mothers, because I truly understand their fears*.” Deepened empathy represented a transformative shift in their professional perspective, moving beyond clinical detachment toward a more profound understanding of the needs and experience of neonatal patients and families.

##### 3.2.3.2. Transformed Care Behaviors and Approaches

Enhanced empathy reshaped nursing practice through behavioral changes, as P10 remarked: “*Previously, I would finish what I was doing first before soothing a crying baby. But now, if he's too fussy, I'll go soothe him first*.” The transformation also involved integrating personal maternal knowledge into professional skills. P11 described applying soothing techniques learned from her own child: “*When they [patients] are fussy…I might try to soothe them to sleep, sometimes playing sounds of amniotic fluid or a hairdryer…I learned these methods from my own baby's experience*.” The overall care approach became more patient-centered, as P14 detailed: “*Now my actions are gentler, I engage in more eye contact and verbal communication with them, and I'm more willing to spend time with these babies*.” These behavioral changes demonstrated enhanced patient-centered practice.

##### 3.2.3.3. Strengthened Responsibility and Evolved Care Philosophy

Maternal identity also reshaped professional responsibility conceptualization, creating deeper commitment to family-centered care. P2 described this change: “*My mentality changed. When you see every baby, you feel their parents have been through a lot. We must be conscientious and responsible…so they can be discharged sooner*.” This was particularly evident in breast milk handling, where maternal experience enhanced professional advocacy and imbued routine practices with new meaning. P13 explained “*Now working in the milk bank, I increasingly feel that we must ensure babies get this milk, because mothers' pumping is also very hard work*.” For participants who personally experienced lactation challenges, this professional duty became even more pronounced. P8 (who experienced pumping difficulties) shared “*When I see other families bringing abundant breast milk, I feel envious. Now I truly cherish their milk-every drop is precious and should be fed to babies*.” P7 illustrated practical application: “*Previously, I might have just discarded unused breast milk, but now I would even use it for oral care if the baby doesn't drink it.*” This evolution in care philosophy reflected a deepened readiness to advocate for both infant welfare and family experiences through a maternal lens.

##### 3.2.3.4. Interplay and Conflict of Dual Roles

The navigation between maternal and professional identities created both synergies in practice and internal conflicts. Professional knowledge informed maternal practice, as P9 described applying hospital routines at home: “*The milk in the ward is warmed to 37 degrees…I felt I was a bit rigidly bringing this hospital habit home*.” Conversely, maternal experiences enriched professional insight. P8 illustrated “*My son also had rhinovirus and suffered greatly. When I see patients with the same condition, I know exactly how uncomfortable they must be*.” However, dual identity navigation also created conflicts requiring ongoing resolution. P7 captured this tension during a painful procedure: “*As a nurse, I have to give him [the neonate] the injection, but as a mother, my thought might be, ‘Don't give it, if you can't get it, then don't*.'” Others noted ethical negotiations, as P6 explained “*As a mother, I don't want to reveal my baby's privacy, but as a nurse, sharing personal experiences can effectively calm families down…I felt it was worth it*.” Despite these tensions, participants ultimately recognized the transformative potential of their dual identity experience. This perspective was powerfully encapsulated by P5's reflection: “*I feel that being a neonatal nurse made me a better mother, and being a mother also made me a better neonatal nurse*,” revealing that maternal and professional identities created a synergistic enhancement rather than irreconcilable tension.

#### 3.2.4. Theme 4. The Ecosystem of Support and Confronting Practical Dilemmas

This theme captured the broader context in which readiness recalibration occurred, revealing how an ecosystem of support and practical workplace realities either facilitated or constrained the transition process. The analysis revealed six key subthemes: foundational family support, valued collegial support, varied managerial support, overwhelming workload and stress, work–family balance struggles, and institutional support gaps and reform needs.

##### 3.2.4.1. Foundational Family Support

Family support systems emerged as essential facilitators of successful transition. P9 emphasized this feeling of being backed: “*My family all encouraged me to go to work and provided strong support…this made me feel very secure about working*.” P17 highlighted practical facilitation: “*My mother-in-law is nice, and my husband helps with the baby after work…taking care of my child is relatively easier because I have helpers*.” Family support also provided emotional security for dual identity navigation. P7 described “*He (my husband) knows about my emotions, and thinks that if I really can't handle it, I should just quit…It feels like he is my backup, that I have a way out*.” Participants perceived this foundational support as the precondition that enabled them to manage the demands of their dual roles.

##### 3.2.4.2. Valued Collegial Support

Peer support proved instrumental in facilitating both technical and emotional aspects of readiness adjustment during the transition. P11 highlighted the importance of colleagues in skill recovery: “*When I first returned, my intravenous indwelling needle puncture skills were rusty…they helped me a lot.*” This support was particularly important in high-stress situations. P5 recalled an emergency admission where colleagues stepped in to take over critical procedures that were part of her duties: “*My colleagues handled the intubation and blood draws…one said to me, ‘Don't worry about this, just get the records done.' That was a huge support.*” Such collegial assistance provided both practical help and emotional reassurance, with P10 describing a colleague's help during a busy time as making her feel “*the world is so beautiful*.” Receiving such peer support was described as crucial for rebuilding confidence.

##### 3.2.4.3. Varied Managerial Support

Leadership responses influenced transition success. Adaptive management was demonstrated by P6: “*When I reported that I couldn't handle the busy C-area due to pumping needs, my supervisor immediately reassigned me to a less busy ward*.” P5 valued symbolic gestures of inclusion: “*Leaders even held a welcome ceremony for us, which makes you feel like you've returned to your family*.” However, supportive leadership varied. P7 described challenging interactions: “*Our leader creates a lot of pressure…when you make a mistake, they make you feel worthless*.” Others reported inadequate emotional acknowledgment or problematic policies, as P17 lamented that “*Emotional support…from the leadership…feels lacking*,” and P10 found her early assignment (2 months postreturn) to night shifts “*very unhappy*.” Managerial approaches varied in their facilitation or constraint of the adjustment process.

##### 3.2.4.4. Overwhelming Workload and Stress

Professional demands created constraints that hindered successful transition. High workload and stress were reported across participants. P7 stated “*Overtime is the norm…there are endless things to do*.” P11 detailed staffing crises: “*We're short-staffed…when I urgently needed help, the charge nurse told me there was no one available…I felt very desperate*.” P15 described night shifts as demanding: “*Many babies, many blood transfusions, and the babies are quite fussy…the work pace is very fast*.” These demands constrained adjustment efforts and contributed to emotional exhaustion, with P7 admitting: “*Overall, I feel extremely overwhelmed and anxious…I consider leaving almost every day*.” High workload conditions challenged returning nurses' capacity for sustained professional practice.

##### 3.2.4.5. Work–Family Balance Struggles

The struggle to balance demanding schedules with family responsibilities complicated the transition process. P7 articulated the tension between personal inclination and professional demands: “*After having a child, I might want to lean more towards my family…But there is so much studying, presentations, exams…I feel pulled in different directions and resistant to these additional demands*.” Such struggles led to feelings of loss and sadness, as P17 shared her anxiety about returning to work: “*The time I get to see him [my baby] every day will be less, which makes me a little sad*.” The emotional toll extended into the workplace, creating a constant mental pull of maternal responsibilities during professional activities. P9 described this feeling: “*During training and meetings…in my mind, I'm just thinking, ‘end quickly, so I can go home and be with my child*.'” These struggles represented ongoing challenges in maintaining balance between the identities as a mother and as a nurse.

##### 3.2.4.6. Institutional Support Gaps and Reform Needs

Participants identified significant gaps between their needs and institutional responsiveness. A critical concern was the lack of adequate breastfeeding support, with P17 stating “*If a fixed time for pumping could be regulated by policy…to ensure milk supply, I think that would be better*.” They also called for more structured re-entry programs, as P1 proposed: “*There should be an adaptation period, starting from lower-acuity wards and gradually adapting before moving to high-acuity wards*.” Additionally, nurses identified a need for more flexible scheduling to improve work–family balance, with P17 expressing desire for “*more consecutive rest days for newly returned mothers*.” These identified gaps revealed opportunities for institutional improvement in supporting nurses' successful return to practice.

## 4. Discussion

This phenomenological study explored the lived experience of neonatal nurses returning to work postpartum. Our findings reveal this transition not as a simple resumption of a previous professional role but as a profound and dynamic process of “readiness recalibration.” This conceptual framework extends beyond existing role transition theories by demonstrating how maternal and professional identities can synergistically enhance, rather than merely compete with, each other, a novel finding that challenges traditional work–family conflict paradigms [35]. Our analysis revealed four interconnected themes that illuminate a sequential transformation process. The establishment of maternal identity (Theme 1) fundamentally reconfigured existing professional readiness, thereby necessitating a multifaceted transition period (Theme 2) characterized by anxiety, physical recovery challenges, and cognitive adjustments. This transition phase served as the transformative catalyst through which maternal experiences were translated into enhanced professional competencies (Theme 3), including deepened empathy and refined care behaviors. The entire recalibration process occurred within an ecosystem that either facilitated or constrained successful adaptation (Theme 4). Unlike conventional role transition models that emphasize role conflict resolution [35, 36], our “readiness recalibration” framework reveals a unique bidirectional enhancement process where maternal role acquisition simultaneously strengthens professional role mastery. As the first study to specifically examine neonatal nurses' postpartum experiences, our findings conceptualize maternal identity as a professional asset rather than impediment and fundamentally reframe understanding of work–family integration in specialized nursing contexts, demonstrating the transformative potential inherent when personal and professional caregiving roles converge.

Consistent with previous studies on nurses in general, participants in our study also faced significant challenges, including work–family conflict, breastfeeding difficulties, and physical and cognitive recovery issues [9–13, 18, 37–40]. These challenges echo Zhou et al.'s [9] findings of “breakdown and healing” among Chinese postpartum nurses and align with Hill et al.'s [12] documentation of heightened stress during emergency nurses' return to work. However, our study extends beyond these adaptation-focused frameworks by revealing how such challenges can transform into professional assets. Our study offers a counter-narrative to the predominantly deficit-oriented literature on nurses' return to work [8]. While existing research frames the postpartum return almost exclusively as a period of conflict and struggle [8, 10, 38, 40, 41], our findings demonstrate that this transition involves both challenges and, more importantly, significant professional growth and enhancement. This is captured in our core finding that “a mother makes a better nurse,” a transformation reflecting what we term “readiness recalibration.” This concept describes a nonlinear adaptation where professional competence is not merely restored but is fundamentally retuned by maternal experience, as evidenced by enhanced empathy, patient-centered care approaches that prioritized patient comfort over task completion, and a strengthened commitment to a family-centered philosophy [42]. Notably, while previous studies have identified stress and adaptation challenges [8, 10, 11], our research uniquely demonstrates how neonatal nursing enables productive integration of maternal and professional identities through direct application of maternal knowledge to professional practice, such as incorporating home soothing techniques learned from personal infant care. These findings suggest that motherhood functions as an informal education for professional development in neonatal nursing.

Furthermore, our findings reveal a specific phenomenon we term empathetic resonance—a double-edged sword unique to neonatal nurses' practice environment. On the one hand, being a new mother created a profound, embodied empathy that drove exceptional compassion for neonatal patients and family. At the same time, empathy created a heightened psychological vulnerability. The intense fear and dread expressed by participants when caring for infant patients with distressing conditions reflected projective maternal anxiety beyond typical clinical empathy [15]. This duality, where the source of enhanced compassionate capacity is also a source of vulnerability, represents a unique psychological challenge for returning neonatal nurses that warrants recognition and support.

Importantly, this recalibration process was deeply embedded within a social and institutional ecosystem. Our findings affirm that the return-to-work experience is not solely an individual's adaptation but a socially mediated process where support is pivotal. This aligns with global research on the importance of social support for working mothers [43–45], but our study reveals a cultural layer that may intensify these dynamics. Specifically, the central role of “foundational family support” observed in our findings reflects traditional Chinese values rooted in Confucian ideals of collective responsibility and intergenerational caregiving, where maternal roles are inherently viewed as supported by broader family networks rather than individual endeavors. This cultural context may also amplify the intense work–family conflict described in our findings, as traditional expectations of maternal devotion and family-centered values can place additional pressure on women to be exemplary mothers, thereby intensifying the stress of balancing demanding professional roles [24, 25]. Ultimately, the ecosystem of support—or lack thereof—from family, colleagues, and managers critically determined whether the recalibration process led to growth or burnout [44, 46], underscoring that supportive environments are essential for successful transitions and that culturally sensitive approaches are vital for effective intervention design.

## 5. Limitations

Several limitations should be acknowledged. Data collection relied on retrospective accounts from the preceding 1 to 12 months, which may involve recall bias; however, interviewing participants at different postreturn time points allowed for a range of perspectives. Recruitment through nurse managers may have introduced selection bias, potentially excluding nurses with severely negative experiences or those who left their positions. This research captured experiences at one point in time; longitudinal studies would provide deeper insights into the temporal dynamics of this transition. Finally, this study focused on nurses' perspectives; future research could benefit from including the perspectives of managers, colleagues, and family members to provide a more holistic understanding of the return-to-work phenomenon.

## 6. Conclusion

This phenomenological study reveals that neonatal nurses' postpartum return to work represents a profound journey of identity transformation and professional readiness recalibration, demonstrating that “a mother makes a better nurse.” The establishment of maternal identity fundamentally transforms nursing practice, creating enhanced empathetic capacity and deeper commitment to family-centered care while simultaneously presenting unique adaptation challenges. Our findings demonstrate that maternal experience, rather than being a professional impediment, serves as a valuable asset that enriches neonatal care. Nevertheless, persistent gaps in institutional support, particularly regarding breastfeeding accommodation and workload management, underscore the need for organizational reform to optimize nurse retention, professional satisfaction, and care quality.

## 7. Implications for Nursing Management

Healthcare organizations should implement evidence-based strategies that recognize returning nurses as valuable assets requiring targeted support rather than remedial intervention. Essential interventions should include structured return-to-work programs that address both clinical skill refreshment and emotional adjustment, alongside robust breastfeeding support through dedicated facilities and protected break times. Organizations should consider implementing flexible work arrangements, including phased-return schedules and temporary exemption from night shifts and high-acuity assignments, to facilitate optimal recalibration. Furthermore, nursing leadership should actively foster supportive environments through empathetic communication while formally recognizing the enhanced compassionate capacity that motherhood brings to practice. These evidence-based organizational strategies can help transform the postpartum return into an opportunity for professional growth while enhancing nurse retention and neonatal care quality. However, realizing the full potential of these strategies requires a tangible commitment to resource allocation, including budgeting for dedicated lactation facilities and appropriate staffing for phased-return schedules, alongside the alignment of institutional policies with national labor and health protections to ensure both feasibility and sustainability.

## Figures and Tables

**Table 1:** Demographic characteristics of participants (*N* = 17).

Characteristics	Category	*n* (%)	Mean (SD)/range
Age (years)			30.18 (2.43) 27–36

Highest education	Master's degree	2 (11.76)	
	Bachelor's degree	12 (70.59)	
	Associate degree	3 (17.65)	

Professional title	Junior	3 (17.65)	
	Intermediate	14 (82.35)	

Years in neonatal nursing (years)			6.35 (2.23)/3–13

Mode of delivery	Vaginal delivery	6 (35.29)	
	Cesarean section	11 (64.71)	

Parity	Primiparous (1 child)	14 (82.35)	
	Multiparous (2 children)	3 (17.65)	

Youngest child's age (months)			9.12 (2.98)/6–14

Transitional work arrangements upon return^1^	Yes	16 (94.12)	
	No	1 (5.88)	

Breastfeeding status at return	Stopped breastfeeding	4 (23.53)	
	Mixed feeding	4 (23.53)	
	Exclusive breastfeeding	9 (52.94)	

Lactation facilities at workplace	Dedicated lactation room	4 (23.53)	
	Private space available	12 (70.59)	
	None	1 (5.88)	

Lactation time at work^2^	Scheduled time	1 (5.88)	
	Time permitted	13 (76.47)	
	No allocated time	3 (17.65)	

Maternity leave duration (months)			6.89 (2.34)/5.30–12.73

Time since return to work (months)			4.90 (3.58)/1.27–10.37

Abbreviation: SD = standard deviation.

^1^Transitional work arrangements include exemption from night shifts during early return period, assignment to lighter duties, or temporary workload reduction.

^2^The categories for “Lactation time at work” are defined as follows: Scheduled time indicates fixed, protected breaks; Time permitted refers to an institutional policy allowing for lactation, but where the timing of breaks is flexible and contingent on the immediate workload of the unit; No allocated time signifies that no specific time was formally designated for lactation by the unit.

## Data Availability

The data that support the findings of this study are available on reasonable request from the corresponding authors. The data are not publicly available due to privacy and ethical restrictions related to the personal nature of the participant interviews.

## Supporting Information

Supporting Table 1. Standards for Reporting Qualitative Research (SRQR) Checklist. Supporting Table 2. Semistructured interview guide. Supporting Table 3. Demographic characteristics of participants.

## References

[1] Ahmad R. S., Sulaiman Z., Nik Hussain N. H., Mohd Noor N. (2022). Working Mothers’ Breastfeeding Experience: A Phenomenology Qualitative Approach. *BMC Pregnancy and Childbirth*.

[2] Okorn A., van Hooff M. L. M., ten Cate A. E. N., Cillessen A. H. N., Beijers R. (2025). Returning to Work After Maternity Leave: a Longitudinal Study Exploring Changes in Postpartum Work Resumption Stress and its Determinants. *Community, Work & Family*.

[3] Franzoi I. G., Sauta M. D., De Luca A., Granieri A. (2024). Returning to Work After Maternity Leave: A Systematic Literature Review. *Archives of Women’s Mental Health*.

[4] Falletta L., Abbruzzese S., Fischbein R., Shura R., Eng A., Alemagno S. (2020). Work Reentry After Childbirth: Predictors of Self-Rated Health in Month One Among a Sample of University Faculty and Staff. *Safety and Health at Work*.

[5] McCardel R. E., Loedding E. H., Padilla H. M. (2022). Examining the Relationship Between Return to Work After Giving Birth and Maternal Mental Health: A Systematic Review. *Maternal and Child Health Journal*.

[6] Alstveit M., Severinsson E., Karlsen B. (2011). Readjusting One’s Life in the Tension Inherent in Work and Motherhood. *Journal of Advanced Nursing*.

[7] Lopez-Gonzalez D. M., Kopparapu A. K. (2025). Postpartum Care of the New Mother. *Statpearls Treasure Island, FL*.

[8] Johnson E., Elder E., Kosiol J. (2025). What are the Experiences of Nurses Returning to Work Following Maternity Leave: A Scoping Review. *BMC Nursing*.

[9] Zhou T., Dong X., Zhang L. (2024). Breakdown and Healing’-Adaptation Experiences of Postpartum Nurses Returning to Work: A Descriptive Phenomenological Study. *BMC Nursing*.

[10] Chen K., Wei L., Zhang Y., Jiang W., Wang J., Pan Y. (2022). Work Stress in Nurses Returning to Tertiary a General Hospitals in China After the Delivery of Their Second Child: A Cross-Sectional Study. *BMC Health Services Research*.

[11] Wan X., Yang J., Pan Y. (2024). Work Experience of Breastfeeding Nurses Returning to Work After Maternity Leave in Liaoning Province of China: A Qualitative Study. *Nursing Open*.

[12] Hill E. K., Bimbi O. M., Crooks N., Brown R., Maeder A. B. (2023). Uncovering the Experience: Return to Work of Nurses After Parental Leave. *Journal of Emergency Nursing*.

[13] Tseng Y. H., Wu K. F., Lin H. R. (2023). Experiences of Female Nurses’ Parental Leave in Taiwan: A Qualitative Study. *Healthcare (Basel)*.

[14] Agency for Healthcare Research and Quality (2025). Nursing and Patient Safety. *Patient Safety Network*.

[15] Shattnawi K. K., Abdallah I. H., Khater W., Alashram S. A. (2021). Experiences of Neonatal Intensive Care Unit Nurses as Mothers of Newborns in Neonatal Intensive Care Units: A Jordanian Qualitative Study. *Journal of Pediatric Nursing*.

[16] Öke Karakaya P., Sönmez S., Aşık E. (2021). A Phenomenological Study of Nurses’ Experiences With Maternal Guilt in Turkey. *Journal of Nursing Management*.

[17] Vilar-Compte M., Hernández-Cordero S., Ancira-Moreno M. (2021). Breastfeeding at the Workplace: A Systematic Review of Interventions to Improve Workplace Environments to Facilitate Breastfeeding Among Working Women. *International Journal for Equity in Health*.

[18] Riaz S., Condon L. (2019). The Experiences of Breastfeeding Mothers Returning to Work as Hospital Nurses in Pakistan: A Qualitative Study. *Women and Birth*.

[19] Ebsco Information Services (2025). Maternal Role Attainment Theory. *Research Starters: Psychology*.

[20] Boniol M., Kunjumen T., Nair T. S., Siyam A., Campbell J., Diallo K. (2022). The Global Health Workforce Stock and Distribution in 2020 and 2030: A Threat to Equity and ‘Universal’ Health Coverage?. *BMJ Global Health*.

[21] National Health Commission of the People’s Republic of China (2025). China Has 5.63 Mln Registered Nurses. https://en.nhc.gov.cn/2024-05/14/c_86312.htm.

[22] Lu H., Hou L., Zhou W. (2021). Trends, Composition and Distribution of Nurse Workforce in China: A Secondary Analysis of National Data from 2003 to 2018. *BMJ Open*.

[23] Zhai Z., Jin G. (2023). China’s Family Planning Policy and Fertility Transition. *Chinese Journal of Sociology*.

[24] Zhao S. (2020). Gender in Families: a Comparison of the Gendered Division of Child Care in Rural and Urban China. *Child and Youth Care Forum*.

[25] Shek D. T. L., Lam C. M., Yang Z. (2019). Division of Labor in Parenting Amongst Chinese Parents in Hong Kong. *International Journal of Child and Adolescent Health*.

[26] Giorgi A. (2009). *The Descriptive Phenomenological Method in Psychology: A Modified Husserlian Approach*.

[27] Paley J. (1997). Husserl, Phenomenology and Nursing. *Journal of Advanced Nursing*.

[28] Fu M. R., Rosedale M. (2009). Breast Cancer Survivors’ Experiences of Lymphedema-Related Symptoms. *Journal of Pain and Symptom Management*.

[29] O’Brien B. C., Harris I. B., Beckman T. J., Reed D. A., Cook D. A. (2014). Standards for Reporting Qualitative Research: A Synthesis of Recommendations. *Academic Medicine*.

[30] Malterud K., Siersma V. D., Guassora A. D. (2016). Sample Size in Qualitative Interview Studies: Guided by Information Power. *Qualitative Health Research*.

[31] Fu M. R., Xu B., Liu Y., Haber J. (2008). Making the Best of it’: Chinese Women’s Experiences of Adjusting to Breast Cancer Diagnosis and Treatment. *Journal of Advanced Nursing*.

[32] Colaizzi P. F., Valle R. S., King M. (1978). Psychological Research as the Phenomenologist Views it. *Existential-Phenomenological Alternatives for Psychology*.

[33] Rusli K. D. B., Ong S. F., Speed S. (2022). Home-Based Care Nurses’ Lived Experiences and Perceived Competency Needs: A Phenomenological Study. *Journal of Nursing Management*.

[34] Lincoln Y. S., Guba E. G. (1986). But is it Rigorous? Trustworthiness and Authenticity in Naturalistic Evaluation. *New Directions for Program Evaluation*.

[35] Greenhaus J. H., Beutell N. J. (1985). Sources of Conflict Between Work and Family Roles. *Academy of Management Review*.

[36] Kramer M. (1974). *Reality Shock: Why Nurses Leave Nursing*.

[37] Castetbon K., Boudet-Berquier J., Salanave B. (2020). Combining Breastfeeding and Work: Findings From the Epifane Population-Based Birth Cohort. *BMC Pregnancy and Childbirth*.

[38] Nichols M. R., Roux G. M. (2004). Maternal Perspectives on Postpartum Return to the Workplace. *Journal of Obstetric, Gynecologic, and Neonatal Nursing*.

[39] Costantini A., Warasin R., Sartori R., Mantovan F. (2022). Return to Work After Prolonged Maternity Leave. An Interpretative Description. *Women’s Studies International Forum*.

[40] Valizadeh S., Hosseinzadeh M., Mohammadi E., Hassankhani H., Fooladi M. M., Cummins A. (2018). Coping Mechanism Against High Levels of Daily Stress by Working Breastfeeding Mothers in Iran. *International Journal of Nursing Science*.

[41] Valizadeh S., Hosseinzadeh M., Mohammadi E., Hassankhani H., M Fooladi M., Schmied V. (2017). Addressing Barriers to Health: Experiences of Breastfeeding Mothers After Returning to Work. *Nursing and Health Sciences*.

[42] Roué J. M., Kuhn P., Lopez Maestro M. (2017). Eight Principles for Patient-Centred and Family-Centred Care for Newborns in the Neonatal Intensive Care Unit. *Archives of Disease in Childhood-Fetal and Neonatal Edition*.

[43] Yuan L., Yumeng C., Chunfen Z., Jinbo F. (2020). Analyzing the Impact of Practice Environment on Nurse Burnout Using Conventional and Multilevel Logistic Regression Models. *Workplace Health & Safety*.

[44] Killien M. G. (2005). The Role of Social Support in Facilitating Postpartum Women’s Return to Employment. *Journal of Obstetric, Gynecologic, and Neonatal Nursing*.

[45] Liu S., Li D., Luo L. (2023). Trajectory of Perceived Social Support on Maternal and its Related Factors-A Longitudinal Research in Southwest China. *Women and Children Nursing*.

[46] Niinihuhta M., Häggman‐Laitila A. (2022). A Systematic Review of the Relationships Between Nurse Leaders’ Leadership Styles and Nurses’ Work-Related Well-Being. *International Journal of Nursing Practice*.

