# Supplementary material for: “A Mother Makes a Better Nurse”: A Phenomenological Study of Neonatal Nurses' Readiness Recalibration Following Postpartum Return to Work
Source: J Nurs Manag. 2025 Nov 2;2025:9095340. doi: 10.1155/jonm/9095340 (PMC12597230; doi:10.1155/jonm/9095340)
Supplement: Supporting Information — Additional supporting information can be found online in the Supporting Information section. [file 9095340.f1.pdf]

Supporting Information

Supporting Table 1. Standards for Reporting Qualitative Research (SRQR) Checklist

No.	Topic	Item	Page
	**Title and abstract**		
S1	Title	Concise description of the nature and topic of the study. Identifying the study as qualitative or indicating the approach (e.g., ethnography, grounded theory) or data collection methods (e.g., interview, focus group) is recommended.	Title page
S2	Abstract	Summary of key elements of the study using the abstract format of the intended publication; typically includes background, purpose, methods, results, and conclusions.	P1
	**Introduction**		
S3	Problem formulation	Description and significance of the problem/phenomenon studied; review of relevant theory and empirical work; problem statement.	P2-3
S4	Purpose or research question	Purpose of the study and specific objectives or questions.	P3
	**Methods**		
S5	Qualitative approach and research paradigm	Qualitative approach (e.g., ethnography, grounded theory, case study, phenomenology, narrative research) and guiding theory if appropriate; identifying the research paradigm (e.g., postpositivist, constructivist/interpretivist) is also recommended; rationale.	P4
S6	Researcher characteristics and reflexivity	Researchers' characteristics that may influence the research, including personal attributes, qualifications/experience, relationship with participants, assumptions, and/or presuppositions; potential or actual interaction between researchers' characteristics and the research questions, approach, methods, results, and/or transferability.	P5-6
S7	Context	Setting/site and salient contextual factors; rationale.	P4
S8	Sampling strategy	How and why research participants, documents, or events were selected; criteria for deciding when no further sampling was necessary (e.g., sampling saturation); rationale.	P4
S9	Ethical issues pertaining to human subjects	Documentation of approval by an appropriate ethics review board and participant consent, or explanation for lack thereof; other confidentiality and data security issues.	P18
S10	Data collection methods	Types of data collected; details of data collection procedures including (as appropriate) start and stop dates of data collection and analysis, iterative process, triangulation of sources/methods, and modification of procedures in response to evolving study findings; rationale.	P5

S11	Data collection instruments and technologies	Description of instruments (e.g., interview guides, questionnaires) and devices (e.g., audio recorders) used for data collection; if/how the instrument(s) changed over the course of the study.	P5 & supporting Table 2
S12	Units of study	Number and relevant characteristics of participants, documents, or events included in the study; level of participation (could be reported in results).	P7 & supporting Table 3
S13	Data processing	Methods for processing data prior to and during analysis, including transcription, data entry, data management and security, verification of data integrity, data coding, and anonymization/deidentification of excerpts.	P5-6
S14	Data analysis	Process by which inferences, themes, etc., were identified and developed, including the researchers involved in data analysis; usually references a specific paradigm or approach; rationale.	P6
S15	Techniques to enhance trustworthiness	Techniques to enhance trustworthiness and credibility of data analysis (e.g., member checking, audit trail, triangulation); rationale*.	P6
	**Results/Findings**		
S16	Synthesis and interpretation	Main findings (e.g., interpretations, inferences, and themes); might include development of a theory or model, or integration with prior research or theory.	P7-14
S17	Links to empirical data	Evidence (e.g., quotes, field notes, text excerpts, photographs) to substantiate analytic findings.	P7-14
	**Discussion**		
S18	Integration with prior work, implications, transferability, and contribution(s) to the field	Short summary of main findings; explanation of how findings and conclusions connect to, support, elaborate on, or challenge conclusions of earlier scholarship; discussion of scope of application/generalizability; identification of unique contribution(s) to scholarship in a discipline or field.	P14-17
S19	Limitations	Trustworthiness and limitations of findings.	P17
	**Other**		
S20	Conflicts of interest	Potential sources of influence or perceived influence on study conduct and conclusions; how these were managed.	P18
S21	Funding	Sources of funding and other support; role of funders in data collection, interpretation, and reporting.	P19

Supporting Table 2. Semi-structured interview guide

Part 1: The Experience of Return-to-Work Adaptation Process

1. Preparation for returning to work: Could you please describe how you prepared as your maternity leave was coming to an end?
   - Probing Question: What were your feelings or state of mind at that time?
   - Probing Question: What kind of conversations or interactions did you have with your family as you prepared to return?

2. Initial return experience: Please describe the situation and your feelings on your first day back at work after maternity leave.
   - Probing Question: How would you describe your physical feelings and emotional state during the initial period of your return?
   - Probing Question: Could you describe a memorable work scene from when you first returned?

3. Readjustment and support: Can you tell me about your experience of re-adapting to the work environment?
   - Probing Question: Who helped you, and in what ways? How did this support make you feel?
   - Probing Question: Could you describe a specific situation where you felt supported, or a situation where you felt a lack of support?

Part 2: The Experience of Maternal Identity in Nursing Practice

4. Nursing practice experience: Please describe a specific situation when you were caring for a baby, and you felt that becoming a mother changed how you were practicing as a nurse in that moment?
   - Probing Question: What was special or different about your nursing actions at that time?
   - Probing Question: What thoughts or feelings related to your maternal identity crossed your mind, and what nursing tasks were you performing simultaneously?

5. Emotional experience: Could you describe a nursing scenario that evoked special emotions for

you after becoming a mother?

- Probing Question: What were your specific feelings and reactions at that moment?

6. Relational experience: Please describe a time when interacting with a patient's family after your return to work made you think of yourself as a mother.
   - Probing Question: What were your feelings or understanding of the parents' emotions and needs at that time?
   - Probing Question: In that scene, when you thought of yourself as a mother, what were your specific interactions with the family like (e.g., what you said, your expressions, or your actions)?

7. Identity experience: Can you describe a specific situation at work that made you particularly aware of being both a nurse and a mother?
   - Probing Question: What exactly happened in that situation? What details left a strong impression on you?
   - Probing Question: What were your physical sensations and emotional reactions at that time?
   - Probing Question: As a nurse, what were you inclined to do or feel? At the same time, as a mother, what were your feelings or thoughts? Did you feel these two states were similar, different, or perhaps in conflict?

Part 3: Reflection and suggestions

8. Looking back on your return-to-work experience, what aspects or feelings were particularly important to you?

9. Are there any other important aspects of your experience as a new mother and a nurse that we haven't talked about yet?

Note: The interview guide provides a framework for the conversation. Core questions open topics, while optional probing questions are used to explore experiences in greater depth. The goal is to follow the participant's narrative and understand their perspective.

Supporting Table 3. Demographic characteristics of participants

Assigned code	Age	Highest education	Professional title	Years in neonatal nursing (years)	Mode of delivery	Parity	Youngest child's age (months)	Transitional work arrangements upon return [1]	Breastfeeding status at return	Lactation facilities at workplace	Lactation time at work [2]	Maternity leave duration (months)	Time since return to work (months)
01	31	Master's degree	Intermediate	6	Cesarean section	Primiparous (1 child)	13	Yes	Stopped breastfeeding	Private space available	No allocated time	5.70	7.90
02	30	Bachelor's degree	Junior	9	Vaginal delivery	Primiparous (1 child)	12	Yes	Exclusive breastfeeding	Private space available	Time permitted	5.70	8.73
03	27	Bachelor's degree	Junior	5	Vaginal delivery	Primiparous (1 child)	8	Yes	Mixed feeding	Dedicated lactation room	Scheduled time	5.90	2.93
04	36	Bachelor's degree	Intermediate	13	Cesarean section	Primiparous (1 child)	7	Yes	Exclusive breastfeeding	Private space available	Time permitted	12.73	2.73
05	31	Associate degree	Junior	8	Cesarean section	Multiparous (2 children)	7	Yes	Exclusive breastfeeding	Private space available	Time permitted	5.57	2.73
06	29	Bachelor's degree	Junior	9	Cesarean section	Primiparous (twins)	8	Yes	Mixed feeding	Private space available	Time permitted	12.50	3.23
07	27	Bachelor's degree	Junior	5	Cesarean section	Primiparous (1 child)	11	Yes	Exclusive breastfeeding	Dedicated lactation room	No allocated time	5.73	6.50
08	29	Bachelor's degree	Junior	7	Vaginal delivery	Primiparous (1 child)	12	Yes	Mixed feeding	Private space available	Time permitted	5.93	10.37
09	33	Bachelor's degree	Junior	10	Cesarean section	Primiparous (1 child)	13	Yes	Stopped breastfeeding	Dedicated lactation room	Time permitted	5.30	9.13
10	30	Bachelor's	Junior	9	Cesarean	Multiparous	14	Yes	Exclusive	Private	Time	5.83	10.67

		degree			section	(2 children)			breastfeeding	space available	permitted		
11	30	Bachelor's degree	Junior	9	Cesarean section	Primiparous (1 child)	12	Yes	Stopped breastfeeding	Private space available	Time permitted	8.77	8.87
12	28	Bachelor's degree	Junior	6	Vaginal delivery	Primiparous (1 child)	7	Yes	Mixed feeding	Private space available	Time permitted	8.20	1.27
13	29	Associate degree	Junior	8	Vaginal delivery	Primiparous (1 child)	7	Yes	Exclusive breastfeeding	Private space available	Time permitted	6.23	1.40
14	33	Bachelor's degree	Junior	11	Cesarean section	Primiparous (1 child)	6	Yes	Stopped breastfeeding	Private space available	Time permitted	5.77	1.97
15	28	Associate degree	Junior	5	Cesarean section	Primiparous (1 child)	6	Yes	Exclusive breastfeeding	Dedicated lactation room	Time permitted	5.70	1.67
16	29	Bachelor's degree	Junior	5	Cesarean section	Primiparous (1 child)	6	Yes	Exclusive breastfeeding	Private space available	Time permitted	5.73	1.93
17	33	Master's degree	Intermediate	10	Vaginal delivery	Primiparous (1 child)	6	No	Exclusive breastfeeding	None	No allocated time	5.80	1.27

Note: [1] Transitional work arrangements include exemption from night shifts during early return period, assignment to lighter duties, or temporary workload reduction. [2] The categories for "Lactation time at work" are defined as: Scheduled time indicates fixed, protected breaks; Time permitted refers to an institutional policy allowing for lactation, but where the timing of breaks is flexible and contingent on the immediate workload of the unit; No allocated time signifies that no specific time was formally designated for lactation by the unit.
